# Supplementary material for: Diverse interactions and ecosystem engineering can stabilize community assembly
Source: Nat Commun. 2020 Jul 3;11:3307. doi: 10.1038/s41467-020-17164-x (PMC7335095; doi:10.1038/s41467-020-17164-x)
Supplement: Supplementary file 1 — Supplementary Information [file 41467_2020_17164_MOESM1_ESM.pdf]

Supplementary Information

Diverse interactions and ecosystem engineering can stabilize community assembly

Yeakel et al.

Supplementary Note 1: Building the source pool

Here and henceforth, we refer to the assembly model presented in the main text as the ENIgMa model (E:eat, N:need, Ig:ignore, Ma:make). To initiate the ENIgMa assembly model, we must first construct the source pool, where each ecological entity (species + modifiers) is defined by their potential interactions. The model is initialized by creating S species and $M = \eta S$ modifiers, such that $N = S + M$ is the expected total number of entities (prior to considering engineering redundancies) and η is the expected number of modifiers made per species in the community, where the expectation is taken across replicates. For each pair of species (x,y) there is a probability p_e that x eats y and probability p_n that x needs y. For each pair of species x and modifier m, there is a probability q_e that species x eats modifier m and a probability q_n that species x needs modifier m. For simplicity we assume throughout that $p_e = q_e$ and that $p_n = q_n$, such that the probability of drawing trophic and service interactions for both species-species and species-modifier interactions is the same.

Without engineering redundancies (i.e. each modifier that a species makes is unique), the expected number of modifiers is $M = \eta S$ where η is the mean number of modifiers made per species S. If we allow for engineering redundancies, the realized number of modifiers $M' < M$. To determine the number of modifiers in the pool, for each species a set number of modifiers is drawn, where $M_i \sim \text{Poiss}(\eta)$. The expected proportion of species that are engineers (species that make modifiers) is thus $1 - e^{-\eta}$, where e is Euler's number. If a particular modifier is randomly and independently drawn for a given engineer from a complete list of all possible modifiers, such that multiple species – with some probability – can make the same modifier, the expected number of modifiers becomes

$$M' = \eta S \left(1 - \frac{1}{e}\right). \tag{1}$$

The frequencies of eat and need interactions, p_e and p_n respectively, are assigned a priori (see Supplementary Supplementary Note 2 for different model parameterizations). The frequency of engineering (make) interactions can be calculated as

$$p_m = \frac{\eta S}{(S + M')^2} = \frac{\eta}{S\left(1 + \eta - \frac{\eta}{\mathrm{e}}\right)^2}. \tag{2}$$

The frequency of the null interaction is then calculated by $p_{\varnothing} = 1 - (p_e + p_n)$ for species-species interactions and $p_{\varnothing} = 1 - (p_e + p_n + p_m)$ species-modifier interactions, respectively. Pairwise interactions are established randomly, such that the source pool matrix has no imbued structure apart from the number of species, the number of modifiers, and the frequency of each directional interaction type. Each source pool is provided a 'basal resource' (the first row/column). A species with a trophic interaction to this resource is identified as an autotroph (or mixotroph depending on its other trophic interactions). If they do not have service dependencies with other species/modifiers, it is these species that are uniquely able to initiate assembly.

When engineering redundancies are allowed, the expected number of unique versus redundant modifiers in the source pool can be determined analytically. The total number of modifiers is $M' = \eta S(1 - \mathrm{e}^{-1})$, and can be subdivided into modifiers that have a unique engineer and those that have multiple engineers. The number of modifiers with a single engineer is $M'_{\text{unique}} = \eta S e^{-1}$. The number of modifiers made by multiple engineers is calculated as $M' - M'_{\text{unique}}$, such that

$$M'_{\text{redundant}} = M' - M'_{\text{unique}} = \eta S \frac{\mathrm{e} - 2}{\mathrm{e}}, \tag{3}$$

such that the proportion of redundant modifiers ϕ is

$$\phi = \frac{M' - M'_{\text{unique}}}{M'} = \frac{\mathrm{e} - 2}{\mathrm{e} - 1} \approx 0.418. \tag{4}$$

Accordingly, we find that the number of redundant modifiers increases linearly with η, while the proportion of modifiers that are redundant is fixed. Figure 8a,b shows both analytical expectations and numerically-derived measures for $M'_{\text{redundant}}$ and ϕ, respectively.

As described in Methods, the assembly process can be simulated efficiently with an event-driven simulation utilizing a Gillespie algorithm. Generally, one computes the rates r_j of all possible events j in a given step. One then selects the time at which the next event happens by drawing a random number from an exponential distribution with mean $1/\sum_j r_j$. At this time, an event occurs that is randomly selected from the set of possible events such that the probability of event a is $r_a/\sum_j r_j$. The effect of the event is then realized and the list of possible events is updated for the next step.

In our framework, at the beginning of each simulation step we compute: 1) all species in the pool and absent from the community that have trophic and service dependencies met by those species in the community: these species are

subject to colonization; 2) all species in the community that do not have a competition strength that is highest for at least one of their resources: these species are subject to primary extinction; 3) all species in the community that do not meet their eat and/or need dependencies: these species are subject to secondary extinction; 4) all modifiers in the community that no longer have an engineer: these modifiers are subject to elimination. We then select one of the four events with a probability proportional to the number of entities that satisfy the criteria for each event. The rates at which each event occurs change at each step, equal to the number of entities that meet the criteria for each event at that point in time. The species/modifier that colonizes or is eliminated from the community is randomly chosen once the event-type is determined.

For example, if the community is empty, and 50 species are able to colonize, the probability of drawing 'colonization' is 1, and the colonizer would be randomly drawn from the 50 capable of colonizing. Another example: if 20 species are able to colonize, 10 species are not superior competitors for any one of their resources, and 30 species do not meet their dependencies, $\sum_j r_j = 60$, and $r_{\text{colonize}} = 1/3$, $r_{\text{primary extinction}} = 1/6$, and $r_{\text{secondary extinction}} = 1/2$. In this case, the most probable event is a secondary extinction. After this single event takes place, the community is updated depending on which event occurred, and the simulation proceeds to the next step. This algorithm is known to offer a much better approximation to the true stochastic continuous time process than a simulation in discrete time steps, while providing a much higher numerical efficiency[1].

Supplementary Note 2: Model parameterizations

Simulations described in the main text have default parameterizations of $S = 200$, $p_e = 0.01$, $c_{\text{n}} = \pi$, $c_{\text{e}} = \sqrt{2}$, $c_{\text{v}} = 1$, and 4000 iterations (time-steps). Replicates are defined as the independent assembly of independently drawn source pools with a given parameterization.

Assembly without ecosystem engineering: Here we set the average number of modifiers made per species $\eta = 0$ and the probability of need interactions in the species pool $p_n = 0.002$.

Structure and dynamics of mutualisms: Again we used the default parameterizations but set $\eta = 0$, while varying $p_n \in [0, 0.002]$. We note that while the density of eat interactions is not changed as service interaction frequency increases, what would previously have been a trophic interaction is more likely to be a mutualism (trophic in one direction, service in the other) if an incoming eat interaction is paired with an outgoing need interaction. In other words, as service interaction frequency increases, eat interactions are not substituted with need interactions (maintaining the fixed trophic interaction frequency), however null interactions are substituted with need interactions.

Assembly with ecosystem engineering: Here we used the default parameterizations but varied $\eta \in [0, 2]$ and $p_n \in [0, 0.002]$.

Supplementary Note 3: Comparison to Niche Model

We compared certain structural features of ENIgMa at steady state to those of the Niche Model[2]. Comparisons were restricted to networks constructed in the absence of engineering because engineers introduce indirect effects that are not considered in static food web models, and may make such comparisons irrelevant. While there are many similarities, there are also some important differences, some of which are highlighted in the main text. While we consider a comparison of our framework with other food web models such as the Niche Model relevant, we emphasize that the motivations underlying both are distinct. Our approach is intended to provide a deeper understanding into how multitype dependencies between species and the environment impact the dynamics of community assembly. While capturing general qualitative features of empirical systems demonstrates that the dynamics we consider are ecologically relevant, the goal of our approach is distinct from that of static food web models, which aim to maximize structural similarities between model and empirical systems[2,3].

We compared steady state ecological networks that emerge from ENIgMa (described in Methods, main text) with food webs constructed from the Niche Model[2] with similar species richness and connectance. Because species richness and connectance of the Niche Model are often altered by eliminating disconnected species, we compared *i*) species richness, *ii*) connectance, *iii*) mean species degree, *iv*) standard deviation of out-degree distributions, and *v*) standard deviation of in-degree distributions averaged across 1000 replicates for each model.

We found that all measures resulted in fairly similar values between ENIgMa and the Niche Model food webs with a some important differences (Figs. 2,3). While similar, ENIgMa produces consistently lower values of connectance, mean species degree, as well as standard deviations of the in- and out-degree distributions. This means that the food webs produced by ENIgMa are more sparsely connected with less variance between species. These results were expected, as the Niche Model assumes systematically increasing dietary ranges with higher niche values, whereas the trophic interactions assigned to species in the source pool of ENIgMa are drawn independently. An important difference between the Niche Model and ENIgMa is that we do not distinguish between traditional consumers and parasites. A different framework known as the Inverse Niche Model[4] has been proposed to address parasitic interactions. The Inverse Niche Model assumes increasing specialization with feeding hierarchies, which would serve to lower the average generality of species (lower degree). In addition, the Inverse Niche model outputs lower standard

deviations of in- and out-degree distributions. Together these trends suggest that the qualitative structural differences that we observe for the assembly and Niche model may reflect an important structural distinction between food webs that do and do not include parasitic species.

Supplementary Note 4: The structure of engineered food webs

We examined whether and to what extent the structure of food webs was altered when engineers are introduced into the community. Because trophic links can now exist between species-modifiers as well as species-species, there are different ways of accounting for structure, making direct comparisons with non-engineered food webs somewhat difficult. We note that we exclude service interactions in this case to best match the structural analysis described in the main text and shown in Fig. 2. While the inclusion of engineers ($\eta = 2$) does have an impact on stability in terms of primary versus secondary extinction rates, there is not a strong effect of engineering on steady state species richness (Fig. 6a; species richness is shown in blue, modifier richness is shown in red).

The role of specialists does and does not change with the introduction of engineering, depending on how specialization is defined. As in the main text, a specialist is defined when its generality index $G_i < 1$ relative to the steady state link density. When engineered modifiers are included, we account for a trophic interaction between a species and another's modifier as an interaction that occurs between those two species indirectly through the modifier intermediary. So if a species B makes a modifier M, and A eats M, then we set A to (indirectly) eat B. This accounting of both direct and indirect trophic interactions between species can then be compared to *i*) the direct trophic link density of the community, or *ii*) the direct + indirect trophic link density of the community, and some insights can be gained from both approaches.

In the first case, where G_i is determined relative to $L^*_{\mathrm{direct}}/S^*$, we find that there are no potential specialists that colonize the community, and (as in the main text) functional specialists colonize first, but (not as in the main text) become functional generalists at steady state (mean proportion specialists at steady state is 0.04; Fig. 6b). This means that the indirect links that define trophic interactions between species and modifiers increase the link-density of the network relative to that defined only by direct trophic interactions. In words, modifiers serve to connect otherwise disconnected species, formalizing the otherwise indirect relationships that structure the role of engineers in the community. In the second case, where G_i is determined relative to $L^*_{\mathrm{indirect}}/S^*$, we find that the changes in both functional and potential specialists over the course of assembly (Fig. 6c) follow those observed for non-engineered food webs (Fig. 2b).

Finally, we observe that while the number of trophic levels increase in the presence of species-modifier interactions, the overall trophic structure of the community advances over the course of assembly in much the same way as it does without engineers (Fig. 6d). Trophic levels are calculated with respect to indirect species interactions through modifier intermediaries. Because species at any trophic level can engineer modifiers used as resources by other species, the mean trophic level of the community is systematically elevated.

Supplementary Figures

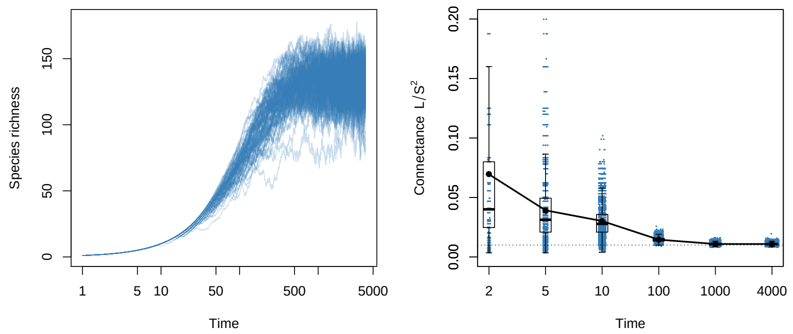

Supplementary Figure 1. Left: Assembly of communities over time results in steady state species richness by ca. time-step 250. Right: Trophic connectance early in assembly is high because a small number of species interact with each other such that the proportion of realized interactions (out of all possible interactions) is closer to unity. Over time, connectance decays as species richness increases, and the density of trophic interactions declines. Boxplots are composed of simulation replicates, with the mean value denoted by the central bar. Box edges define the 25th and 75th percentiles of simulation results, and whiskers represent 1.5× the interquartile range.

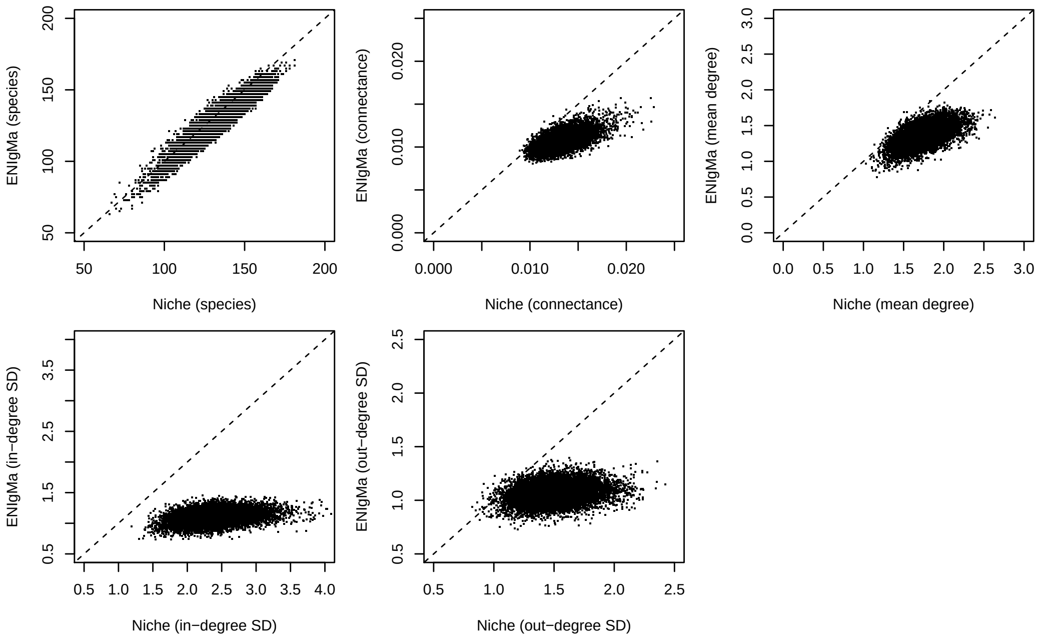

Supplementary Figure 2. Comparisons of raw structural measures for the assembly (y-axis) and Niche model (x-axis). If the models produce similar structures, metrics will tend to fall on the 1:1 line (drawn). While the values for both models are similar, connectance, mean degree, and the standard deviation of in- and out-degree are all lower for the assembly model relative to those measures for the Niche model.

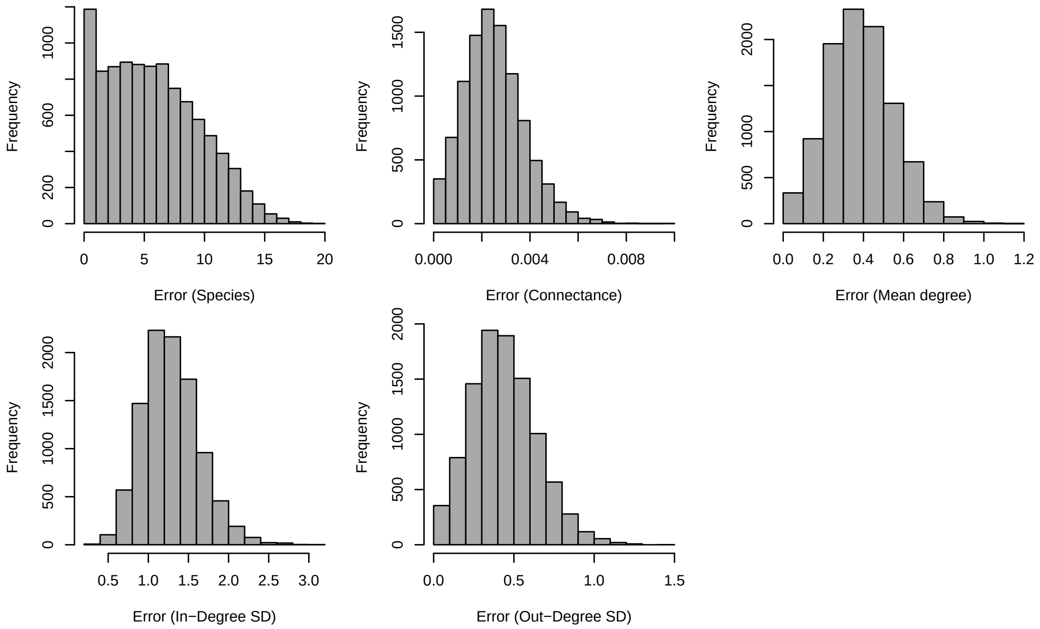

Supplementary Figure 3. Error between structural measures of the assembly and Niche models. Error is measured as $\sqrt{(m_i - m_j)^2}$, where m_i and m_j are structural metrics for the assembly and Niche model, respectively. Only the trophic network of the assembly model was used to assess metrics.

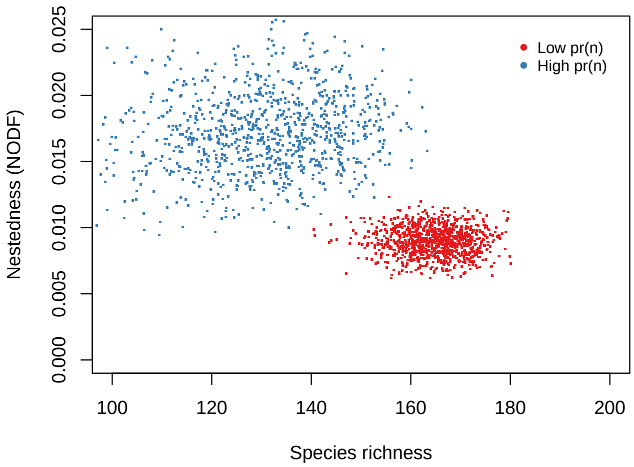

Supplementary Figure 4. Nestedness (UNODF) as a function of steady state richness for 1000 replicated communities without service interactions ($p_n = 0$) compared to those with a high frequency of service interactions ($p_n = 0.002$). While higher frequencies of service interactions do lower steady state species richness (due to increasing secondary extinction rates), there is not a relationship between nestedness and species richness across replicates for a given service interaction frequency.

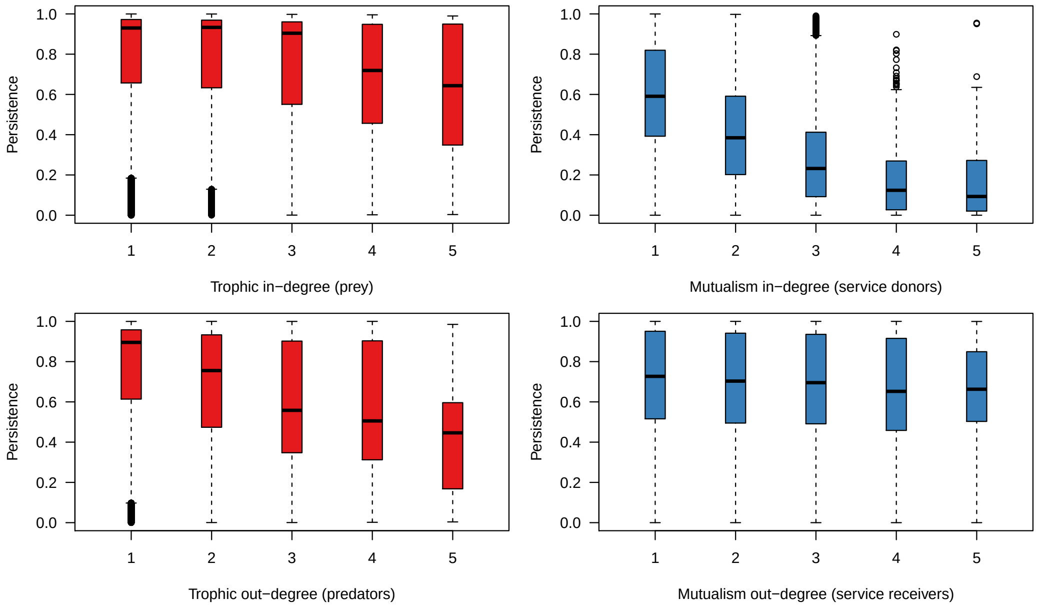

Supplementary Figure 5. Persistence as a function of trophic and mutualism in/out-degree for communities with higher densities of service interactions ($p_e = 0.01$; $p_n = 0.002$). Left column: species-specific persistence as a function of trophic in-degree (the number of prey a species has; top) and out-degree (the number of predators a species has; bottom). Right column: species-specific persistence as a function of the mutualism in-degree (the number of service donors a species has; top) and out-degree (the number of service receivers a species has; bottom). As the trophic in- and out-degree of species increases, competition strength is lowered and persistence decreases. As the mutualism in-degree increases, so does the number of service donors that are needed for the receiving species to remain in the community. This introduces structural constraints that lowers persistence. Boxplots are composed of simulation replicates, with the mean value denoted by the central bar. Box edges define the 25th and 75th percentiles of simulation results, and whiskers represent 1.5$\times$ the interquartile range.

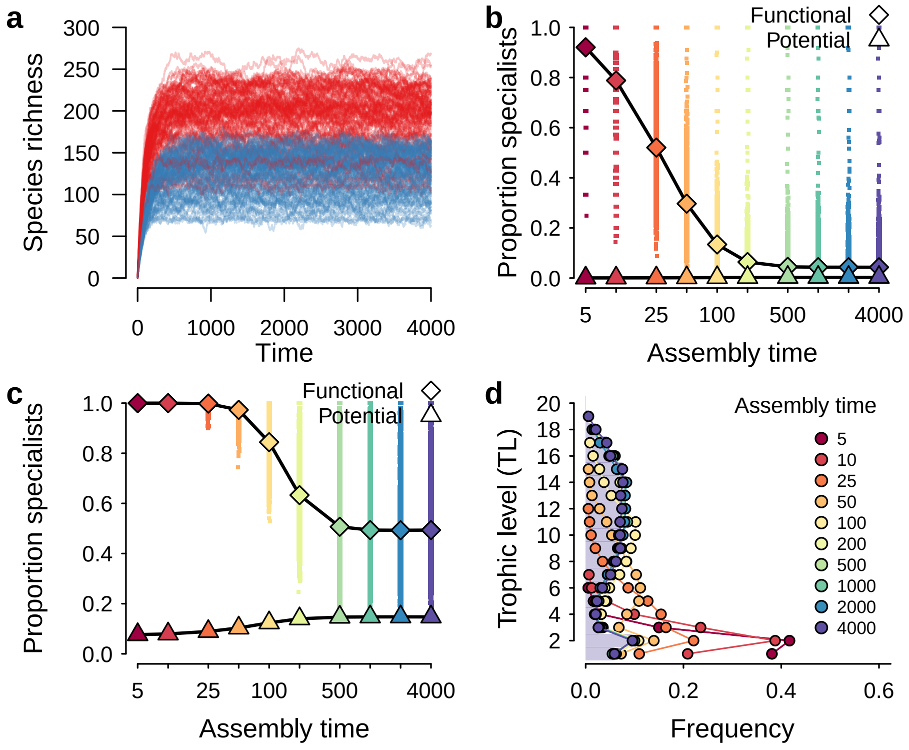

Supplementary Figure 6. **a**, Assembling communities over time from a pool of 200 species, some of which are ecosystem engineers and produce modifier nodes. Species richness is blue; modifier richness is red. Steady state species richness is reached by $t = 250$. **b**, The proportion of specialists as a function of assembly time, where a specialist is defined as a species with a generality index $G_i < 1$ relative to the steady state link density. Here G_i is scaled to the steady state link density where links are direct trophic interactions between species. Diamonds represent functional (realized) trophic interactions; triangles represent potential trophic interactions. **c**, The proportion of specialists as a function of assembly time, where a specialist is defined as a species with a generality index $G_i < 1$. Here G_i is scaled to the steady state link density where links are composed of both direct trophic interactions between species and indirect trophic interactions between consumers and those species that produce modifiers as resources. Diamonds represent functional (realized) trophic interactions; triangles represent potential trophic interactions. **d**, The frequency distribution of trophic levels as a function of assembly time (iterations). Autotrophs occupy $\mathrm{TL} = 1$. Measures were evaluated across 10^4 replicates; see Methods for parameter values.

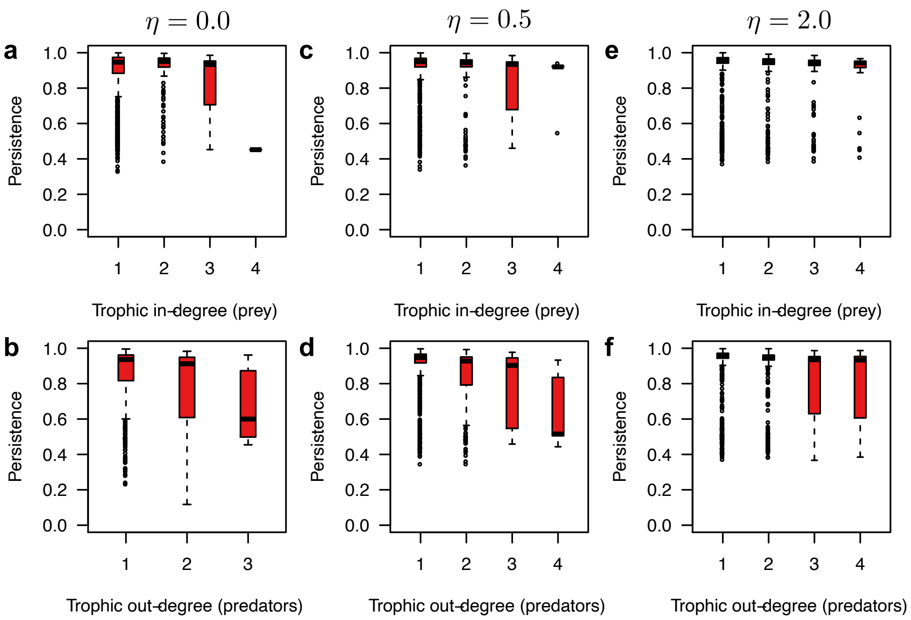

Supplementary Figure 7. Species-specific persistence as a function of **a**, trophic in-degree (number of resources a species has; top) and **b**, out-degree (number of consumers that eat the species; bottom) when there are no engineers in the community. Species-specific persistence as a function of **c**, trophic in-degree (number of resources a species has; top) and **d**, out-degree (number of consumers that eat the species; bottom) when engineers are rare ($\eta = 0.5$). The notion that having a small number of engineers and modifiers in the community increases rates of primary extinction (Fig. 4a) by stabilizing consumers at the expense of their prey is supported by *i*) increased persistence of generalist consumers, and *ii*) the presence of species with larger number of predators. Species-specific persistence as a function of **e**, trophic in-degree (number of resources a species has; top) and **f**, out-degree (number of consumers that eat the species; bottom) when engineers are common ($\eta = 2.0$). The notion that a large number of engineers and modifiers in the community decrease rates of primary extinction (Fig. 4a) due to expanding niche space (diffusing the effects of competitive exclusion) is supported by the lack of correlation between trophic in/out-degree and persistence. Boxplots are composed of simulation replicates, with the mean value denoted by the central bar. Box edges define the 25th and 75th percentiles of simulation results, and whiskers represent 1.5× the interquartile range.

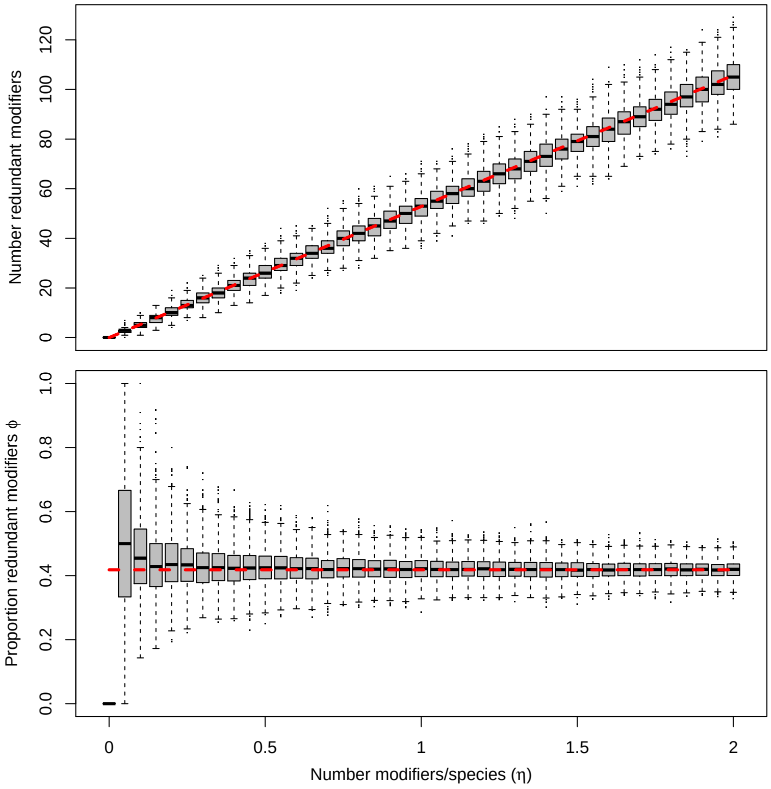

Supplementary Figure 8. **a**, Number of redundant modifiers in the source pool as a function of the expected number of modifiers made per species η. The red dashed line shows the analytical expectation (Supplementary Equation (3)). **b**, Proportion of redundant modifiers ϕ versus the total number of modifiers in the source pool as a function of the expected number of modifiers made per species η. The red dashed line shows the analytical expectation of $\phi \approx 0.418$ (Supplementary Equation (4)). Boxplots are composed of simulation replicates, with the mean value denoted by the central bar. Box edges define the 25th and 75th percentiles of simulation results, and whiskers represent $1.5\times$ the interquartile range.

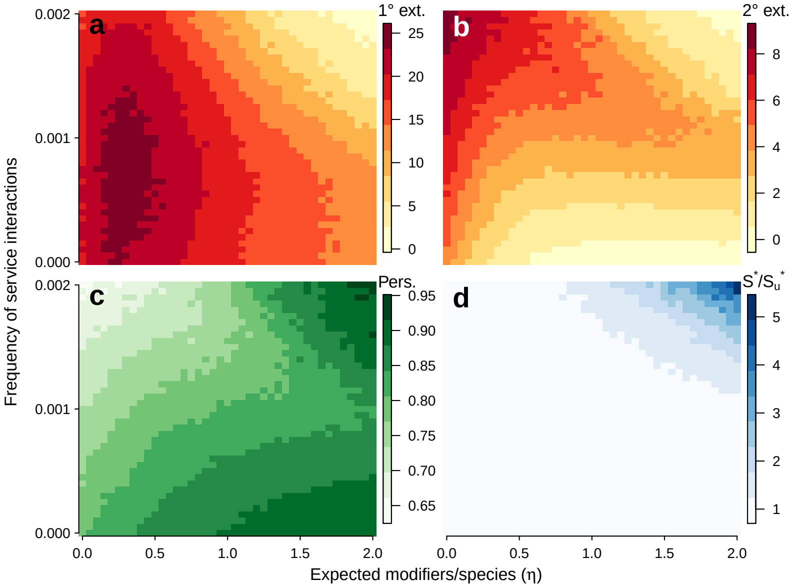

Supplementary Figure 9. Measures of community stability as a function of the frequency of service interactions and number of modifiers per species, where each modifier is uniquely made by an engineer. **a**, Mean rates of primary extinction, where primary extinctions occur from competitive exclusion of consumers over shared resources. **b**, Mean rates of secondary extinction, which cascade from primary extinctions. **c**, Mean species persistence, defined as the percent simulation time the community is occupied by a given species, averaged across all species that successfully colonize. **d**, The ratio S^*/S^*_{u}, where S^*_{u} denotes steady states for systems where all engineered modifiers are unique to each engineer, and S^* denote steady states for systems with redundant engineering. Higher values of S^*/S^*_{u} mean that systems with redundant engineers have higher richness at the steady state than those without redundancies. Primary and secondary extinction rates were evaluated at the community level, whereas persistence was determined for each species and averaged across the community. Each measure reports the expectation taken across 50 replicates. See Methods and Supplementary Supplementary Note 2 for parameter values.

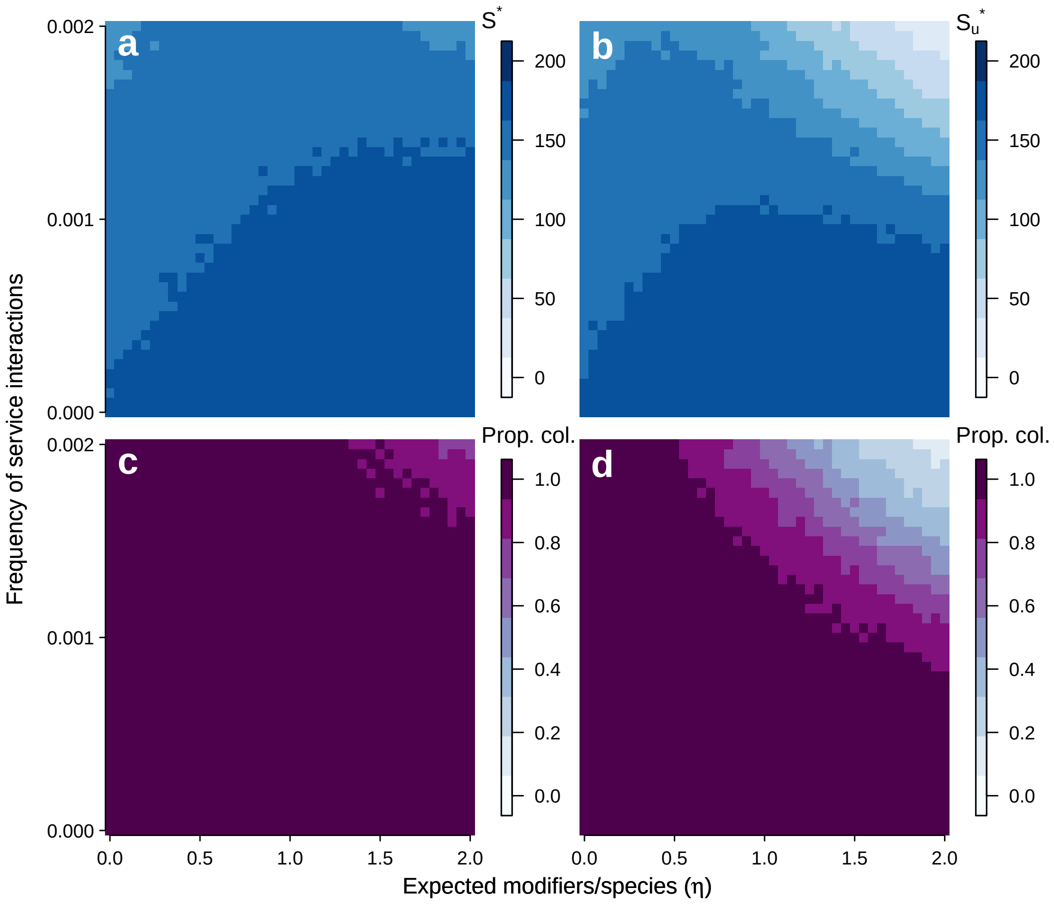

Supplementary Figure 10. **a**, Steady state community richness with redundant engineering. **b**, Steady state community richness without redundant engineering. **c**, Proportion of species in the source pool that colonize the community at least once throughout the simulation (with redundant engineering). **d**, Proportion of species in the source pool that colonize the community at least once throughout the simulation (without redundant engineering).

Supplementary References

[1] Gillespie, D. T. Exact stochastic simulation of coupled chemical reactions. *J. Phys. Chem.* **81**, 2340–2361 (1977).

[2] Williams, R. J. & Martinez, N. D. Simple rules yield complex food webs. *Nature* **404**, 180–183 (2000).

[3] Williams, R. J. & Purves, D. W. The probabilistic niche model reveals substantial variation in the niche structure of empirical food webs. *Ecology* **92**, 1849–1857 (2011).

[4] Warren, C. P., Pascual, M., Lafferty, K. D. & Kuris, A. M. The inverse niche model for food webs with parasites. *Theor. Ecol.* **3**, 285–294 (2010).
